# Analysis of Biomechanical Parameters of Muscle Soleus Contraction and Blood Biochemical Parameters in Rat with Chronic Glyphosate Intoxication and Therapeutic Use of C_60_ Fullerene

**DOI:** 10.3390/ijms22094977

**Published:** 2021-05-07

**Authors:** Dmytro Nozdrenko, Olga Abramchuk, Svitlana Prylutska, Oksana Vygovska, Vasil Soroca, Kateryna Bogutska, Sergii Khrapatyi, Yuriy Prylutskyy, Peter Scharff, Uwe Ritter

**Affiliations:** 1 Department of Biophysics and Medical Informatic, Taras Shevchenko National University of Kyiv, 01601 Kyiv, Ukraine; ddd@univ.kiev.ua (D.N.); psvit_1977@ukr.net (S.P.); vmsoroka@gmail.com (V.S.); bogutska_ki@knu.ua (K.B.); prylut@ukr.net (Y.P.); 2 Lesya Ukrainka Volyn National University, 43025 Lutsk, Ukraine; Abramchuk.Olga@vnu.edu.ua; 3 National University of Life and Environmental Science of Ukraine, 03041 Kyiv, Ukraine; 4 Bogomolets National Medical University of Kyiv, 01601 Kyiv, Ukraine; ovvigovskaya@gmail.com; 5 Interregional Academy of Personnel Management, 03039 Kyiv, Ukraine; khrapatiysv@gmail.com; 6 Institute of Chemistry and Biotechnology, Technical University of Ilmenau, 98693 Ilmenau, Germany; peter.scharff@tu-ilmenau.de

**Keywords:** glyphosate, C_60_ fullerene, muscle soleus of rat, biomechanical parameters, blood biochemical parameters

## Abstract

The widespread use of glyphosate as a herbicide in agriculture can lead to the presence of its residues and metabolites in food for human consumption and thus pose a threat to human health. It has been found that glyphosate reduces energy metabolism in the brain, its amount increases in white muscle fibers. At the same time, the effect of chronic use of glyphosate on the dynamic properties of skeletal muscles remains practically unexplored. The selected biomechanical parameters (the integrated power of muscle contraction, the time of reaching the muscle contraction force its maximum value and the reduction of the force response by 50% and 25% of the initial values during stimulation) of muscle soleus contraction in rats, as well as blood biochemical parameters (the levels of creatinine, creatine phosphokinase, lactate, lactate dehydrogenase, thiobarbituric acid reactive substances, hydrogen peroxide, reduced glutathione and catalase) were analyzed after chronic glyphosate intoxication (oral administration at a dose of 10 μg/kg of animal weight) for 30 days. Water-soluble C_60_ fullerene, as a poweful antioxidant, was used as a therapeutic nanoagent throughout the entire period of intoxication with the above herbicide (oral administration at doses of 0.5 or 1 mg/kg). The data obtained show that the introduction of C_60_ fullerene at a dose of 0.5 mg/kg reduces the degree of pathological changes by 40–45%. Increasing the dose of C_60_ fullerene to 1 mg/kg increases the therapeutic effect by 55–65%, normalizing the studied biomechanical and biochemical parameters. Thus, C_60_ fullerenes can be effective nanotherapeutics in the treatment of glyphosate-based herbicide poisoning.

## 1. Introduction

Glyphosate (N-(phosphonomethyl) glycine) is a non-selective herbicide most commonly used for weed control. Among herbicides, it ranks first in the world in production. Many agricultural crops are genetically engineered to tolerate glyphosate. This significantly increases the effectiveness of weed control in these crops. The effect of glyphosate on a plant is due to the fact that it inhibits the components of the enzyme system of the shikimate pathway of biosynthesis of benzoic aromatic compounds [1]. Animals do not have such an enzyme system and therefore this herbicide is considered to be relatively harmless to them. The half-lethal dose (LD_50_) of glyphosate is >5 kg/kg of rat body weight with a single administration [1]. 

In recent years, there has been a growing worldwide concern about the possible direct and indirect health effects of the widespread use of glyphosate. In 2015, the World Health Organization reclassified glyphosate as likely carcinogenic to humans [2]. There is considerable controversy regarding its carcinogenicity and toxicity, with very different opinions of the scientists and regulatory bodies involved in the glyphosate study. One of the key aspects of this controversy is the extent of pathological changes in laboratory animals that are caused by glyphosate [3]. The most convincing data indicate that glyphosate causes hemangiosarcomas, tumors and malignant lymphomas, renal and liver adenomas, nervous and miotic disorders of a wide spectrum of severity [3].

Glyphosate also causes numerous morphological, physiological and biochemical disorders in the cells and organs of animals, including mammals. It worsens the condition of the gastrointestinal tract: a violation of its contractile function is observed already at a concentration of 3 mg/L; the violation of motility continues after the removal of glyphosate from the incubation solution [4]. The use of glyphosate as a herbicide in agriculture can lead to the presence of its residues and metabolites (aminomethylphosphonic acid) in food for human consumption and thus pose a threat to human health. The authors [5] found that glyphosate reduces energy metabolism in the brain, its amount increases in white muscle fibers. 

At the same time, the effect of chronic use of glyphosate on the dynamic properties of skeletal muscles remains practically unexplored. It was shown that the activity of acetylcholinesterase (AChE) did not change in the muscles and brain of animals exposed to glyphosate during the first 96 h. On the contrary, the expression of this enzyme in muscle tissue changed [6]. The consequence of these pathological processes is disorders in the dynamics of contraction of the muscular system of varying severity. The results [7] show that glyphosate intoxication increases energy expenditure to maintain homeostasis. In particular, there was a decrease in the level of glycogen and triglycerides in all organs and an increase in lipid peroxidation (LPO). 

The mechanisms for the toxicity of glyphosate-based drugs are complex. It is difficult to separate the toxicity of glyphosate from the toxicity of the drug as a whole, or to determine the contribution of surfactants to the overall toxicity. As a result, the treatment of poisoning occurs for a long time, symptomatic and ineffective [8]. 

Studies of the effect of a glyphosate-based herbicide on AChE enzyme activity and oxidative stress at concentrations of 0.5–10.0 mg/L for 96 h followed by an equal recovery period indicate the presence of LPO and AChE inhibition. The results also showed an increased level of thiobarbituric acid reactive substances (TBARS) at all tested herbicide concentrations, which remained elevated even after a recovery period [9]. According to the authors, the triggering mechanisms of the onset of these pathological cascades are associated precisely with the formation of a large number of free radicals. They initiate LPO, cause direct inhibition of mitochondrial enzymes of the respiratory chain and their ATPase activity, inactivation of glyceraldehyde 3-phosphate dehydrogenase and membrane sodium channels. 

The ability of C_60_ fullerenes to inactivate free radicals was described back in 1991 [10]. One C_60_ molecule simultaneously captures 34 methyl radicals, effectively inactivates the superoxide anion radical and hydroxyl radicals in vitro system, protecting cell membranes from oxidation [11]. It is assumed that biocompatible and water-soluble C_60_ fullerenes [12] can be considered as powerful scavengers of free radicals during the development of ischemia and fatigue processes in skeletal muscle [13,14]. In our previous works on in vivo models, it was shown that the usage of safe doses of water-soluble C_60_ fullerene at the initiation of various pathologies leads to significant positive therapeutic effects, in particular, during acute liver injury, colorectal cancer, obesity, acute cholangitis and hemiparkinsonism [15,16,17,18,19,20]. 

Based on the above data, the purpose of this work was to estimate the therapeutic effect of water-soluble C_60_ fullerene, as a powerful antioxidant, on the development of muscle pathologies in rat skeletal muscle caused by chronic glyphosate intoxication. 

## 2. Results and Discussion

### 2.1. AFM Analysis

It is known that the size of C_60_ fullerene particles in aqueous solution strongly correlates with their specific biological properties and toxicity. So, the antibacterial activity of C_60_ fullerene is connected with its ability to undergo aggregation [21]; the macrophage apoptosis induced by aqueous C_60_ fullerene aggregates changes the mitochondrial membrane potential [22]; the respiratory toxicity and immunotoxicity of C_60_ fullerenes in mice and rats after nose inhalation strictly depends on their nano- and micro-size [23]; depending on the size C_60_ fullerenes can inhibit BK_Ca_ but not K_v_ channels in pulmonary artery smooth muscle cells [24], penetrate through plasma membrane inside the cell [25] or be adsorbed on the surface of the membrane [26]. Therefore, the size effect of C_60_ fullerene particles in aqueous solution is considered now to be very important. 

**Figure 1:**
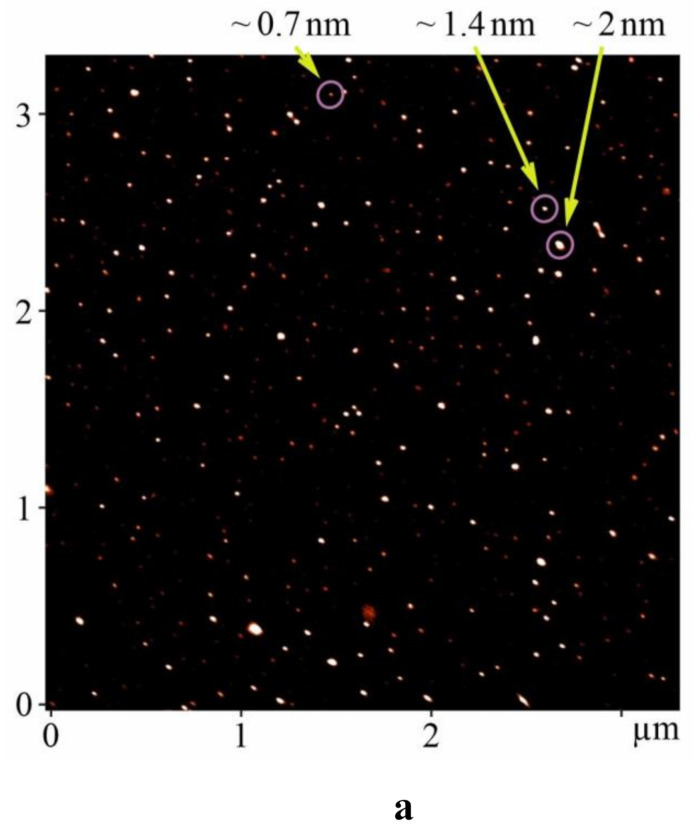

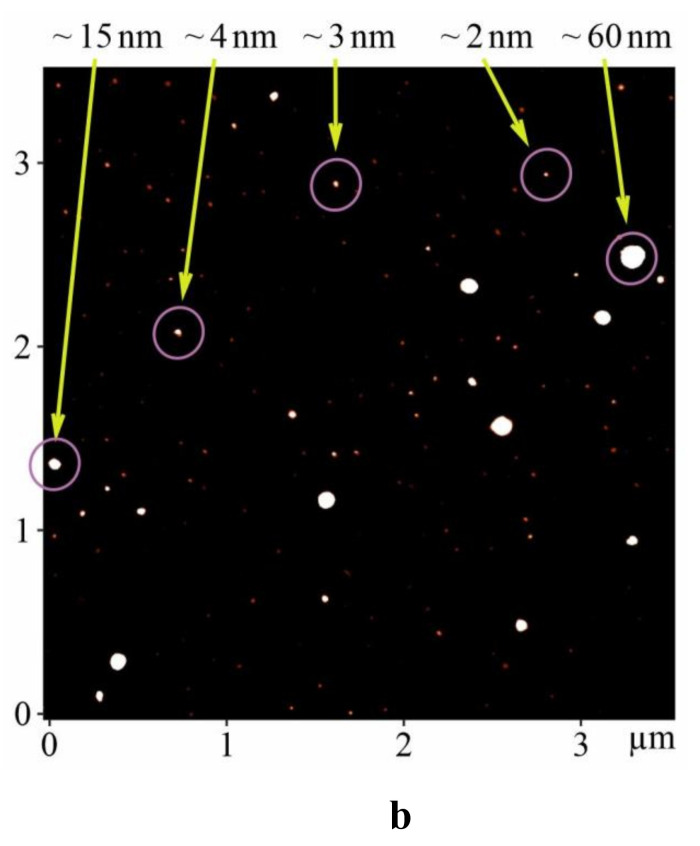
AFM images (tapping mode) of C_60_ fullerene nanoparticles on the mica surface (concentration 0.15 mg/mL) (**a**) objects with a height of ~0.7 nm (**b**) objects with a height of 1.4–60 nm.

The atomic force microscopy (AFM) study of C_60_ fullerene films deposited from an aqueous solution revealed a high degree of molecules dispersion in solution. It turned out that C_60_ fullerene aqueous solution (C_60_FAS) contains both single C_60_ fullerene (see the objects with a height of ~0.7 nm in Figure 1a) and its labile nanoaggregates (objects with a height of 1.4–60 nm in Figure 1b). The majority of C_60_ molecules were located chaotically and separately along the surface, or in the form of bulk clusters. Thus, C_60_FAS is a polydisperse colloid nanofluid. This result is in a good agreement with our previous probe microscopic data [27,28].

In addition, the stability of the used C_60_FAS was evaluated by the zeta potential measurement. This value was shown to be −30.3 mV at room temperature. Such a high (by absolute value) zeta potential for the C_60_FAS indicates its high stability (low tendency for nanoparticle aggregation over time) and suitability for further biological research. 

### 2.2. Biomechanical Analysis

**Figure 2:**
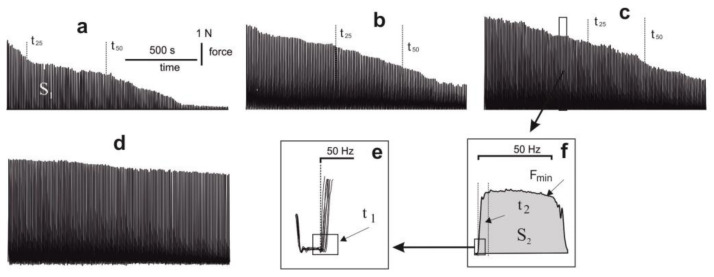
Curves of the generation of the contraction force of muscle soleus rat after chronic intoxication with glyphosate for 30 days: (**a**), (**b**), (**c**) and (**d**)—the curves of muscle contraction for 1500 s with the administration to the animals of glyphosate, glyphosate and C_60_FAS at doses of 0.5 and 1 mg/kg, respectively, and with the administration to the animals of distilled water (control group); (**e**) mechanograms of single contractions; (**f**) an example of calculating the time of the onset of a muscle response. S_1_ is the integrated power of muscle contraction throughout the entire period of stimulation; S_2_ is the integrated power in a single contraction; F_min_ is the minimum value of force generation in a single contraction; t_50_ and t_25_ are the time of decreasing the maximum force response to 50% and 25% of the initial amplitude of muscle force; t_1_ and t_2_ are the time of the onset of the muscle response and the force reaching its maximum value in a single contraction.

**Figure 3:**
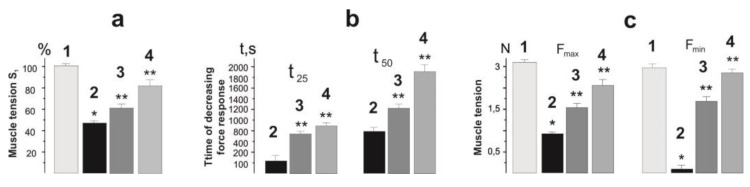
Parameters of contractile activity of muscle soleus rat after chronic intoxication with glyphosate for 30 days: (**a**) integrated power of muscle contraction throughout the entire period of stimulation (S_1_), presented as a percentage of control values; (**b**) time of decreasing the force response by 50% (t_50_) and 25% (t_25_) from the initial values; (**c**) maximum (F_max_) and minimum (F_min_) fixed forces of muscle contraction throughout the entire duration of stimulation. 1—control group (native muscle); 2—the glyphosate group; 3—the glyphosate+C_60_ fullerene (0.5 mg/kg) group; 4—the glyphosate + C_60_ fullerene (1 mg/kg) group; * *p* < 0.05 relative to the control group; ** *p* < 0.05 relative to the glyphosate group.

In the process of analysis of the force curves obtained during stimulation of muscle soleus by 5 s pools for 1500 s after chronic intoxication of animals with glyphosate for 30 days, serious disorders in muscle dynamics are visible (Figure 2). The integrated power of muscle contraction during the whole period of stimulation decreased to 41 ± 3% of the control values (Figure 3). A significant reduction in the force response ended in complete muscle rigidity after 1200 s. However, in animals treated with C_60_FAS, this parameter was 57 ± 2% and 68 ± 4% at doses of C_60_ fullerene 0.5 and 1 mg/kg, respectively. It should be noted that in this case, the muscle responded with a contractile response throughout the stimulation period, not falling below 30% of the limit (Figure 2 and Figure 3). The time of reduction of the force response by 50% and 25% from the initial values increased from 103 ± 11 s and 790 ± 17 s after glyphosate poisoning to 760 ± 8 s and 1213 ± 14 s and 940 ± 21 s and 1820 ± 24 s after therapeutic use of C_60_FAS at doses of 0.5 and 1 mg/kg, respectively. The maximum and minimum recorded forces of muscle contraction throughout stimulation were 0.81 ± 0.10 N and 0.30 ± 0.05 N after glyphosate poisoning, 1.65 ± 0.20 N and 1.72 ± 0.20 N and 2.67 ± 0.30 N and 2.93 ± 0.30 N after therapeutic use of C_60_FAS at doses of 0.5 and 1 mg/kg, respectively (Figure 2 and Figure 3). 

**Figure 4:**
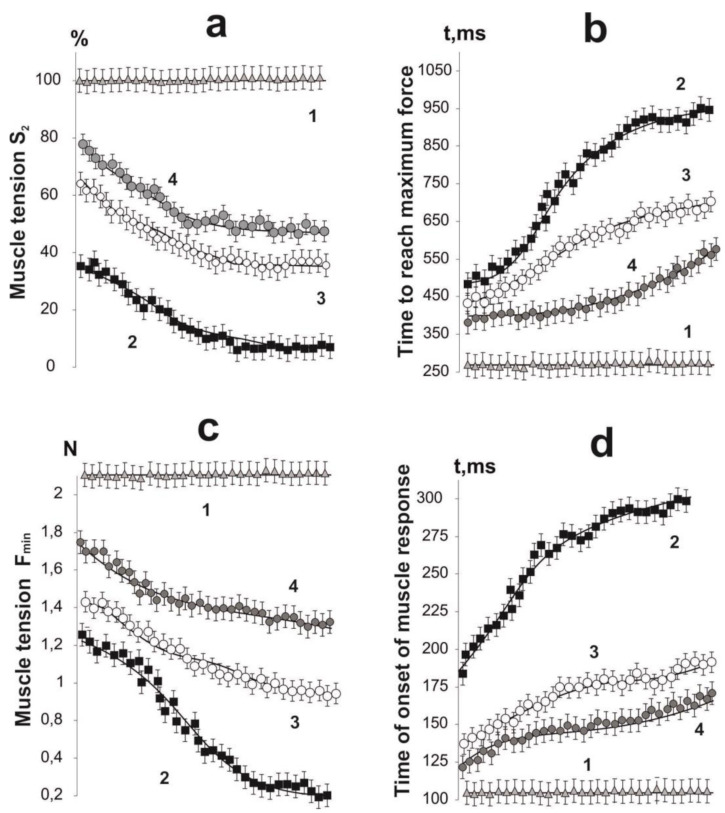
Parameters of single contractions of muscle soleus rat after chronic intoxication with glyphosate for 30 days, caused by 5 s stimulation with a frequency of 50 Hz: (**a**) integrated muscle power (S_2_), calculated from the total area of the force curves as a percentage of the control values; (**b**) time to reach the maximum force response; (**c**) minimum (F_min_) fixed force of muscle contraction; (**d**) time of onset of muscle response to stimulation. 1—control group (native muscle); 2—the glyphosate group; 3—the glyphosate+C_60_ fullerene (0.5 mg/kg) group; 4—the glyphosate+C_60_ fullerene (1 mg/kg) group.

A decrease in the strength activity of a muscle during glyphosate poisoning can be explained by a violation of energy metabolism. So, in a study [29], the authors found that a high concentration of the herbicide led to a significant decrease in the energy reserve in the muscles, showing an unfavorable sublethal effect on energy metabolism and, consequently, on the dynamic properties of the muscular system in general. The recorded significant positive therapeutic effect of C_60_ fullerene may be associated exclusively with its antioxidant properties, which reduce the degree of damage to cell membranes. To confirm this, we analyzed the biomechanical parameters of single muscle contractions (Figure 4). 

The dynamics of the contractile component is determined by the sensitive mechanisms of interaction of motor neuron pools with actin and myosin myofilaments. The influence of pathological factors on these processes leads either to a complete dysfunction of this process, or to its desynchronization. As a result, the whole muscle, as a dynamic system, is unable to adequately implement the pools of neural activity coming from the central nervous system (CNS). The nature and level of such dysfunctions is directly related to the level of development of pathological processes in both muscle and nervous tissue. The results of studies [30] show that the effect of the glyphosate-based herbicide affects the CNS of rats, possibly altering the neurotransmitter systems that regulate locomotor activity. 

The experiment made it possible to trace the therapeutic effect of C_60_FAS on different regions of the generation of the force response of the rat muscle after chronic intoxication with glyphosate (Figure 4). A change in the time the force reaches its maximum level is one of the most important parameters of the kinetics of skeletal muscle contraction. This component of muscle dynamics is especially important in controlling hand contraction in humans. Pathological processes occurring in the nervous or muscle tissue lead to its increase, which complicates, and in some cases completely blocks the possibility of accurate positioning of the joint with the damaged muscle [31]. After taking glyphosate for 30 days, this parameter increased significantly. It should be noted that this increase was progressive with growing in the number of contractions: from 470 ± 27 ms with the first contraction to 954 ± 33 ms with the last contraction (in control, 250 ± 11 ms). These values changed significantly after the therapeutic use of C_60_FAS: with a dose of C_60_ fullerene of 0.5 mg/kg, this time was 430 ± 22 ms and 650 ± 29 ms, respectively, and with a dose of 1 mg/kg—367 ± 19 ms and 543 ± 24 ms, respectively (Figure 4). Thus, the protective effect of C_60_FAS was more than 30% in the first and more than 65% in the second cases. 

To understand the features of muscle dynamics during the development of a pathological process, it is important to analyze the rate of processing of stimulation pools emanating from the CNS into the mechanical component of contraction and the possibility of modifying the kinetics of contraction under the influence of pathological changes. A change in the time of the onset of muscle response after nerve stimulation is one of the most important parameters of the kinetics of skeletal muscle contraction. Analysis of the data obtained showed a significant increase in this parameter after glyphosate poisoning from 175 ± 22 ms with the first contraction to 298 ± 27 ms with the last compared with the control—102 ± 8 ms. C_60_FAS therapy at a dose of C_60_ fullerene 0.5 mg/kg reduced this time to 132 ± 19 ms and 184 ± 17 ms during the first and last muscle contractions, respectively, and with a dose of C_60_ fullerene 1 mg/kg—to 120 ± 20 ms and 165 ± 14 ms, respectively. It should be noted that significant decrease in this indicator under the action of both doses of C_60_ fullerene (Figure 4): the protective effect of C_60_FAS was more than 65% in the first case and more than 75% in the second case. 

A change in the level of minimum force of muscle contraction generation is an indicator of significant changes caused by pathological processes in the myocyte. This indicator is not associated with neuropathic damage and its analysis gives an idea of violations of the force generation system within the muscle fiber. When performing fairly simple single-joint movements, this marker is the main indicator of muscle dysfunction, the phenomenological analysis of which makes it possible to establish the presence of causal relationships between the levels of decrease in the biomechanical activity of muscles and the development of the pathological process [32]. With a constant level of the minimum force of more than 2 N in the control, its drop with the use of glyphosate ranged from 1.3 ± 0.1 N to zero. C_60_FAS therapy increased the level of the minimum force of muscle contraction to (1.4–0.9) ± 0.1 N at a dose of C_60_ fullerene 0.5 mg/kg and up to (1.7–1.4) ± 0.1 N at a dose of C_60_ fullerene 1 mg/kg, respectively. In this case, the protective effect of C_60_FAS was more than 50% in the first case and more than 75% in the second case. 

All these changes ultimately lead to a change in the overall strength activity of the muscle, which can be quantified by the value of the integrated power. A change in this parameter can be associated with disorder in both neural component and muscular component of the studied pathology [33]. With chronic use of glyphosate, the integrated power decreased from 41 ± 3% to zero. C_60_FAS therapy brought this level to 63 ± 3%—47 ± 5% at a dose of C_60_ fullerene 0.5 mg/kg and 80 ± 6%—54 ± 2% at a dose of C_60_ fullerene of 1 mg/kg, respectively. The protective effect of C_60_FAS was more than 50% in the first case and more than 60% in the second case. 

### 2.3. Biochemical Analysis

**Figure 5:**
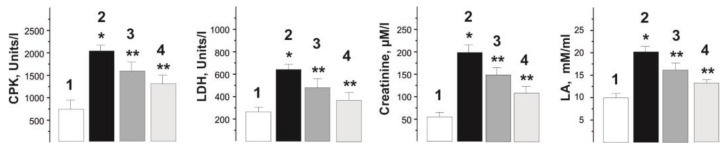
Biochemical parameters of rat blood (CPK, LDH, creatinine and LA) after chronic glyphosate intoxication for 30 days. 1—control group (native muscle); 2—the glyphosate group; 3—the glyphosate+C_60_ fullerene (0.5 mg/kg) group; 4—the glyphosate+C_60_ fullerene (1 mg/kg) group; * *p* < 0.05 relative to the control group; ** *p* < 0.05 relative to the glyphosate group.

Analysis of biochemical markers of rat blood, in particular creatinine, creatine phosphokinase (CPK), lactate (LA) and lactate dehydrogenase (LDH), makes it possible to assess the physiological changes occurring in skeletal muscle and the effect of a therapeutic drug on pathological processes in it. Studies have shown that the levels of the selected markers have a pronounced tendency to increase in the blood of rats intoxicated with glyphosate and decrease during C_60_FAS therapy (Figure 5). 

The change in the concentration of CPK, an enzyme from the energy supply system of musculoskeletal cells, from 756 ± 26 U/L in the norm to 1950 ± 33 U/L after glyphosate intoxication, in our opinion, may be the result of destruction of myocyte walls caused by the influence of the herbicide, with partial release of intramyocytic enzymes into the extracellular space. With the use of C_60_FAS, the CPK level decreased by 23.2 ± 3% and 31.7 ± 2% at doses of C_60_ fullerene 0.5 and 1 mg/kg, respectively (Figure 5). 

Analysis of changes in the level of LDH made it possible to assess the overall health of the injured muscle. The increase in the LDH level after administration of glyphosate increased from 254 ± 13 U/L (normal) to 659 ± 26 U/L and is evidence of the development of significant dysfunctions of the neuromuscular drug and, as a consequence, the development of fatigue processes. After therapeutic use of C_60_FAS, the LDH level decreased by 27 ± 3% and 31 ± 2% at doses of C_60_ fullerene 0.5 and 1 mg/kg, respectively. 

The change in creatinine level from 50 ± 2 µM/L in the control to 196 ± 4 µM/L with chronic intake of glyphosate confirms previously obtained data that increased serum creatinine level is an important factor for predicting the severity of glyphosate poisoning [34]. C_60_FAS therapy led to a significant decrease in its levels to 157 ± 3 µM/L and 112 ± 4 µM/L at doses of C_60_ fullerene 0.5 and 1 mg/kg, respectively. In our opinion, the decrease in the creatinine fraction in this case is caused by the antioxidant properties of C_60_ fullerene, its ability to reduce inflammatory reactions and protect the membranes of skeletal muscle cells from nonspecific free radical destruction by efficient absorption of free radicals [35]. 

Contraction of skeletal muscles leads to the accumulation of LA and H^+^ ions and, accordingly, to acidification of the intra- and extracellular media, which reduces the production of ATP and suppresses the activity of Na^+^, K^+^-ATPase. This leads to a delay in the generation of action potentials and reduces muscle activity. Pathological processes in the myocyte increase this imbalance towards acidification of the medium and, thus, the LA level is an important marker for assessing the degree of muscle activity. Analysis of the LA level showed its increase from 10 ± 1 mM/mL (normal) to 19 ± 2 mM/mL after using glyphosate. The use of C_60_FAS therapy reduced its level to 16 ± 2 mM/mL and 14 ± 1 mM/mL at doses of C_60_ fullerene 0.5 and 1 mg/kg, respectively. 

Glyphosate is an endocrine disruptor in chronic ingestion, exhibiting high cytotoxicity. The previously obtained results [36] show that it affects survival due to deregulation of the cell cycle and metabolic changes that can alter mitochondrial oxygen consumption, increase free radical levels, damage DNA, cause hypoxia, accumulation of mutations and, ultimately, cell death. It was also shown that after exposure to the herbicide for 8 days at a concentration of 0.95 mg/L, there was an increase in the amount of TBARS in muscle and brain tissues. An increase in reduced glutathione (GSH) level also indicated a compensatory response of the body against toxic conditions. Oxidative stress that arose during the period of exposure to the herbicide was probably caused by increased LPO [30]. Thus, a change in the level of endogenous antioxidants is an important marker that determines the degree of physiological disorders in muscle cells during glyphosate intoxication. 

**Figure 6:**
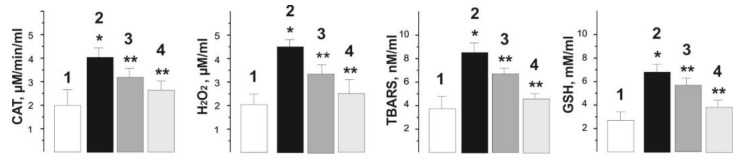
Indicators of pro- and antioxidant balance (CAT, H_2_O_2_, TBARS and GSH) in the blood of rats after chronic intoxication with glyphosate for 30 days. 1—control group (native muscle); 2—the glyphosate group; 3—the glyphosate+C_60_ fullerene (0.5 mg/kg) group; 4—the glyphosate+C_60_ fullerene (1 mg/kg) group; * *p* < 0.05 relative to the control group; ** *p* < 0.05 relative to the glyphosate group.

Figure 6 shows the results of measurements of indicators of pro- and antioxidant balance in the blood of experimental rats. The data obtained indicate increased levels of peroxidation and oxidative stress as well as endogenous antioxidants with the use of the herbicide. The increase in these biochemical markers compared to control values was 218 ± 19%, 251 ± 14%, 280 ± 19% and 250 ± 24% for TBARS, hydrogen peroxide (H_2_O_2_), GSH and catalase (CAT) activity, respectively. 

The level of these markers decreased significantly after therapeutic use of C_60_FAS. So, the TBARS level decreased to 170 ± 11% and 120 ± 8% of the control values, H_2_O_2_—160 ± 14% and 114 ± 11%, GSH—150 ± 12% and 128 ± 9%, CAT activity—140 ± 14% and 119 ± 10% at doses of C_60_ fullerene 0.5 and 1 mg/kg, respectively. 

Summarizing, the proposed therapy with the use of low doses of water-soluble C_60_ fullerenes, possessing membranotropic [25,37] and powerful antioxidant properties [38], leads to positive biomechanical and biochemical changes in the character of contractile processes in the skeletal muscles of rats with chronic glyphosate intoxication. 

## 3. Materials and Methods

To obtain C_60_FAS (maximum concentration 0.15 mg/mL), a method based on the transfer of these carbon molecules from toluene to water followed by sonication was used [27,39]. The prepared C_60_FAS was stored at a temperature of +4 °C for 12 months. 

The AFM (Solver Pro M system, NT-MDT, Moscow, Russia) was performed to determine the size of C_60_ fullerene particles in the prepared aqueous solution. A drop of investigated solution was transferred on the atomic-smooth substrate to deposit layers. Measurements were carried out after complete evaporation of the solvent. For AFM study, a freshly broken surface of mica (SPI supplies, V-1 grade) was used as a substrate. Measurements were carried out in a semicontact (tapping) mode with AFM probes of the RTPESPA150 (Bruker, 6 N/m, 150 kHz) type. 

The zeta potential was measured to assess the stability of the prepared C_60_FAS using the Zetasizer Nano-ZS90 technique (Malvern, Worcestershire, UK). 

The experiments were performed on male Wistar rats aged 3 months weighing 170 ± 5 g. The study protocol was approved by the bioethics committee of Taras Shevchenko National University of Kyiv in accordance with the rules of the European Convention for the Protection of Vertebrate Animals Used for Experimental and Other Scientific Purposes and the norms of biomedical ethics in accordance with the Law Of Ukraine №3446—IV 21.02.2006, Kyiv, on the Protection of Animals from Cruelty during medical and biological research. 

In total, 40 rats divided into four groups (10 animals each) were used in the study. Glyphosate was administered daily at a dose of 10 μg/kg of animal weight orally using a metal catheter for 30 days (*n* = 10). The animals of the control group (*n* = 10) were injected with an equivalent volume of distilled water for 30 days. C_60_FAS was administered at doses of 0.5 (*n* = 10) and 1 mg/kg of animal weight (*n* = 10) immediately after administration of the herbicide for 30 days. Measurements of the studied parameters (see below) in all groups were performed on the 31^st^ day after the start of the experiment.

It should be noted that the use of selected doses of C_60_FAS are based on previous experimentally established data, which showed a high protective effect of water-soluble C_60_ fullerenes [13,14,19]. Additionally, it should be noted that the doses of C_60_ fullerene used in our experiments are significantly lower than the LD_50_ value, which was 600 mg/kg body weight when administered orally to rats [40] and 721 mg/kg when administered intraperitoneally to mice [25]. 

Anesthesia of animals was performed by intraperitoneal administration of nembutal (40 mg/kg). Preparation of the experiment included the cannulation (*a. carotis communis sinistra*) for the therapeutic administration of the drug and pressure measurement, tracheotomy and laminectomy at lumbar spinal cord level. Muscle soleus of rat was released from the surrounding tissues. Its tendon was cut across in distal part, which was connected to the force sensors. For modulated stimulation of efferents, the ventral roots were cut at the points of their exit from the spinal cord. Stimulation of efferents was performed by electrical pulses lasting 2 ms, generated by the generator, through platinum electrodes. The control of the external load on the muscle was performed using a system of mechanical stimulators. Perturbation of the load was carried out by a linear electromagnetic motor [41]. 

The choice of muscle soleus for this study is due to the fact that this muscle contains the maximum number of slow fibers, which is important for accurate and high-quality fixation of fast-acting processes, occurring in the anterior front of the tetanus, in pathology.

To induce muscle contraction, a stimulation signal with a frequency of 50 Hz and a duration of 5 s was used without a relaxation period. The total duration of stimulation was 1500 s. The current strength, at which the muscle began to contract, was considered a threshold, and further stimulation was performed with a current strength of 1.3–1.4 thresholds. 

To record the force of skeletal muscle contraction, we used the original strain gauge that consists of force and length sensors, a synchronous pulse generator and a thermal control system [13]. 

In the process of analyzing the obtained results, the following parameter was used: the integrated power of muscle contraction (calculated area under the force curve), which is an indicator of the overall performance of the muscle with the applied stimulation pools. The development of muscle contractile activity was assessed by calculating the time of the decrease in the force response by 50% and 25% of the initial values during stimulation. We also analyzed the time to reach the maximum value of the muscle contraction force and the delay in the onset of the muscle response. 

The level of enzymes content in the blood of experimental animals (creatinine, CPK, LA, LDH, TBARS, H_2_O_2_, GSH and CAT), as marker of muscle injury [42], was determined using clinical diagnostic equipment—a haemoanalyzer [13]. 

Statistical processing of results was performed by methods of variation statistics using software Original 9.4. We conducted at least six repetitions for each measurement. Data are expressed as the means ± SEM for each group. The differences among experimental groups were detected by one-way ANOVA followed by Bonferroni’s multiple comparison test. Values of *p* < 0.05 were considered significant.

## 4. Conclusions

The obtained results indicate that the therapeutic administration of water-soluble C_60_ fullerenes at a dose of 0.5 mg/kg reduces the degree of pathological changes in rats caused by chronic glyphosate intoxication by 40–45%. Increasing the dose of water-soluble C_60_ fullerenes to 1 mg/kg increases the therapeutic effect by 55–65%, normalizing the studied biomechanical and biochemical parameters. Considering the fact that poisoning with glyphosate compounds has a lethality of up to 20% and there is currently no antidote to them, and the basis for the treatment of systemic toxicity is deactivation and aggressive supportive therapy [34], the proposed C_60_ fullerene therapy of this type of intoxication opens up new prospects for clinical trials. 

## Data Availability

Not applicable.
